# Microplastics and 17α Ethinylestradiol: How Do Different Aquatic Invertebrates Respond to This Combination of Contaminants?

**DOI:** 10.3390/toxics12050319

**Published:** 2024-04-28

**Authors:** Caio Rodrigues Nobre, Beatriz Barbosa Moreno, Aline Vecchio Alves, Mayana Karoline Fontes, Bruno Galvão de Campos, Leticia Fernanda da Silva, Luciane Alves Maranho, Luís Felipe de Almeida Duarte, Denis Moledo de Souza Abessa, Rodrigo Brasil Choueri, Paloma Kachel Gusso-Choueri, Camilo Dias Seabra Pereira

**Affiliations:** 1 Department of Marine Sciences, Federal University of São Paulo, Santos Campus (UNIFESP—Santos), Rua Maria Máximo, 168, Santos 11030-100, Brazil; beatrizbarbosa3@gmail.com (B.B.M.); alinee.vecchioalves@gmail.com (A.V.A.); rodrigo.choueri@unifesp.br (R.B.C.); camilo.seabra@unifesp.br (C.D.S.P.); 2 Biosciences Institute, São Paulo State University (UNESP), Litoral Paulista Campus, Praça Infante Dom Henrique, s/n, Parque Bitaru, São Vicente 11330-900, Brazil; mayanakf@gmail.com (M.K.F.); bruno12323@hotmail.com (B.G.d.C.); leticia-fernanda.silva@unesp.br (L.F.d.S.); denis.abessa@unesp.br (D.M.d.S.A.); 3 Morphofunctional Laboratory, University of Ribeirão Preto (UNAERP), Avenida Dom Pedro I, 3.300, Guarujá 11440-003, Brazil; lmaranho@gmail.com; 4 Department of Ecotoxicology, Santa Cecília University (UNISANTA), Rua Oswaldo Cruz, 266, Santos 11045-907, Brazil; duarte.mepi@gmail.com (L.F.d.A.D.); palomakachel@unisanta.br (P.K.G.-C.)

**Keywords:** emerging pollutants, pharmaceuticals, synthetic hormones, oyster, crab, toxicity

## Abstract

The synthetic hormone 17α ethinyl estradiol (EE2) is a molecule widely used in female contraceptives and recognized as a contaminant of attention (Watch List) in the European Union due to its high consumption, endocrine effects and occurrence in aquatic environments. Its main source of introduction is domestic sewage where it can be associated with other contaminants such as microplastics (MPs). Due to their characteristics, they can combine with each other and exacerbate their isolated effects on biota. This study evaluated the combined effects of microplastics (MPs) and 17α ethinylestradiol (EE2) on two tropical estuarine invertebrate species: *Crassostrea gasar* and *Ucides cordatus*. Polyethylene particles were spiked with EE2 and organisms were exposed to three treatments, categorized into three groups: control group (C), virgin microplastics (MPs), and spiked microplastics with EE2 (MPEs). All treatments were evaluated after 3 and 7 days of exposure. Oysters exhibited changes in phase 2 enzymes and the antioxidant system, oxidative stress in the gills, and reduced lysosomal membrane stability after exposure to MPs and MPEs. Crabs exposed to MPs and MPEs after seven days showed changes in phase 1 enzymes in the gills and changes in phases 1 and 2 enzymes in the hepatopancreas, such as disturbed cellular health. The combined effects of microplastics and EE2 increased the toxicity experienced by organisms, which may trigger effects at higher levels of biological organization, leading to ecological disturbances in tropical coastal ecosystems.

## 1. Introduction

The synthetic hormone 17α ethinylestradiol (EE2), a molecule commonly used in female contraceptives, is a synthetic estrogen derived from the natural estrogen 17β estradiol that is used in human medicine, veterinary medicine, and aquaculture. Due to its high use, EE2 has become a substance of great concern since its presence is detected in surface waters around the world, occurring in concentrations that vary between 6.6 and 27.7 ng/L^−1^, which has resulted in EE2 being added to the list of priority pollutants to be monitored by the European Union Watch List for Water Emerging Pollutants in 2018 [1,2,3,4]. EE2 possesses a high logKoW (3.6–4.2) and persists in marine environments; it has a half-life of more than 20 days in water and sediment [5]. EE2 is frequently reported in wastewater, surface water, sediment, and marine biota; even when found at low concentrations, it is capable of producing adverse effects on organisms [6,7].

Prior studies have reported isolated effects of EE2 on different estuarine and marine organisms, including gastropods, amphipods, mysids, shrimp, urchins, and bivalves [8,9,10]. When present in domestic sewage, EE2 can interact with other pollutants, including microplastics.

Microplastics are particles (<5 mm) composed of a variety of polymers. It is estimated that there are around 268,940 tons of plastic particles drifting in the oceans, of which 92.4% are microplastics [11,12].

Microplastics are able to interact with a variety of substances, particularly those hydrophobics such as EE2. Through sorption processes, microplastics often serve as carriers and dispersers of other molecules in marine ecosystems [13,14,15,16]. Combining these molecules may have adverse effects on the biota through direct ingestion or indirectly through the release of adsorbable substances [17,18,19,20]. 

Previous studies have described the association between microplastics and steroid hormones [21,22]; however, studies that evaluate the effects of this combination on the biota are scarce, particularly in terms of estuarine animals. Estuaries are frequently contaminated by domestic wastewater and port effluents, sources of EE2 and microplastics that increase the coastal biota’s risk of exposure to these pollutants. 

Hydrophobic pollutant detoxification involves a series of enzymes typically divided into phase 1 (biotransformation) and phase 2 enzymes (conjugation). This process may also generate different intermediary metabolites, such as reactive oxygen species. Changes to aquatic organisms’ antioxidant defense systems may be the consequence of an increase in reactive oxygen species synthesis induced by exposure to xenobiotics and may lead to oxidative stress when mechanisms of defense are overtaken by pro-oxidant forces. In this situation, adverse effects such as DNA strand breaks, enzyme inactivation, lipid peroxidation, and protein or lipid degradation may occur.

Among the estuarine species susceptible to such changes as a result of exposure to these xenobiotics are the oyster *Crassostrea gasar* and the crab *Ucides cordatus*.

Popularly known as the mangrove oyster, *Crassostrea gasar* is an estuarine species that occurs on the Brazilian coast from the State of Pará to Santa Catarina [23]. They are widely used for ecotoxicological studies, as they are filter-feeding organisms and have a sessile life habit and bioaccumulation capacity; this species has been used in several studies using biomarkers [24,25,26,27], demonstrating that they are excellent sentinel organisms to achieve the true characterization of environmental impacts on aquatic ecosystems.

The brachyurian crab *U. Cordatus* occurs from the state of Florida, in the United States, to the state of Santa Catarina, in Brazil [28]. This species is among the main mangrove species, whose ecological relevance is due to the cycling of nutrients resulting from its feeding process through the consumption of litter [29]. Due to this fact and importance, *U. cordatus* has been adopted as a biological model in conservation and impact studies on the mangrove ecosystem [30,31,32]. In this sense, this study sought to assess adverse effects of EE2-spiked polyethylene microparticles by evaluating phase 1 and 2 enzymes, the antioxidant defense system, and lipid peroxidation, DNA strand breaks, cholinesterase activity, and lysosomal membrane stability in two estuarine species (*Crassostrea gasar* [the mangrove oyster] and *Ucides cordatus* [the swamp ghost crab]) in order to better understand the impacts of this combination of pollutants on tropical coastal zones. These species were chosen because of their local ubiquity, ecological relevance to mangrove ecosystems, socioeconomic significance, and their viability for ecotoxicological assays [18,32,33].

## 2. Materials and Methods

Linear low-density polyethylene (LLDPE) particles (microplastics) between 100 and 250 µm in size, used as abrasives in cosmetic products (kindly provided by Braskem S/A), were contaminated using a 1 mg·L^−1^ EE2 solution following the method described by Nobre et al. [18]. A total of 22.5 g of polyethylene microparticles was added to 1 L of EE2 solution at a nominal concentration of 1 mg·L^−1^; this concentration was used for greater adsorption of the substance on the particles and better understanding of the mechanistic effects that the EE2 carried by microplastics can have on organisms.

The sample was then poured into a vial, protected from light, and placed on an orbital shaker at 250 rpm for 48 h to reach sorption equilibrium. Subsequently, the microspheres were removed using Grade GF/C glass microfiber filters (Whatman), stored in amber glass bottles, and placed in cooling chambers until the exposure period.

After the procedure, aliquots of virgin and EE2-spiked microplastics were analyzed using high-performance liquid chromatography (HPLC) combined with a mass spectrometer to determine the real concentration of the pharmaceutical under study.

The microplastic sample’s polymeric confirmation was conducted using a Fourier transform infrared spectrophotometer (FT-IR), specifically the Perkin Elmer Spectrum Two model equipped with the Universal Attenuated Reflectance (ATR) accessory. Analysis was carried out using Spectrum 10 software with the following settings: IR range spanning from 4000 cm^−1^ to 550 cm^−1^, data range set to 1 cm^−1^, resolution adjusted to 4 cm^−1^, and 16 accumulation scans performed. 

The specimens were exposed to three treatments. The control treatment contained seawater with dimethyl sulfoxide (the solvent used to prepare the EE2 stock solution used in microplastic contamination); the virgin microplastic (MP) treatment consisted of 250 mg·L^−1^ (∼2.75 × 10^7^, items m^−3^) of uncontaminated microplastics; and the EE2 (MPE) treatment relied on 250 mg·L^−1^ of microplastics contaminated by EE2. 

The concentration of 250 mg·L^−1^ was adopted to better understand the effects of MPs alone or in association with EE2. This was based on a previous study by Siegfried et al. [34], in which, using a predictive model, they estimated the release of 510 mg·L^−1^ of plastic microspheres into the Atlantic Ocean through European seas in the year 2050.

The *C. gasar* used in the experiment were obtained from a farm located within the Mandira extractive reserve located in the city of Cananéia, São Paulo State, Brazil. The U. cordatus crabs were collected from mangrove swamps in the Barra do Una region of the Juréia-Itatins Marine Protected Area, located in the city of Peruíbe, São Paulo State, Brazil (SISBIO authorization: 60314-1). 

In the experiments, the oysters were taken to the laboratory and acclimated in a 350 L tank in seawater at salinity 30‰ for 7 days. The tank included a filtration system and controlled physical and chemical parameters. The organisms were fed a 4–10 µm phytoplankton suspension (Phytogold-S—Brightwell) every 48 h. After the acclimation period, ten specimens were selected from each treatment. The crabs were acclimated in 500 L tanks with 50 L of seawater at salinity 30‰ for 10 days. The physical and chemical parameters (pH, dissolved oxygen, salinity, and ammonia) in the tank were monitored with the aid of a multiparametric probe, and the crabs were fed with *Rhizophorae mangle* leaves obtained from the same sampling site. 

After the acclimatization period, the organisms were subjected to treatments for 3 and 7 days, with 10 organisms per treatment per time subsequently removed. For the analysis of biomarkers, the gills, hepatopancreas, muscles, and hemolymph of 7 individuals of each species were collected. In addition, digestive gland samples were collected from oysters, and hepatopancreas tissue samples were collected from crabs. The collected tissues were placed in cryotubes and stored at −80 °C until the analyses were carried out. At the end of each exposure period, water, microplastic, and 3 organism samples were also obtained and stored at −20 °C for further chemical analyses to determine EE2 concentrations in the different matrices. 

In order to measure EE2 in the different samples, the EPA Method 1694 for solid phase extraction was used [35].

Analytical measurements were performed using high-performance liquid chromatography (HPLC) combined with a mass spectrometer (Agilent 1260 series, Agile Technologies, QTRAP 3200, AB Sciex, São Paulo, Santos, Brazil). The column used was an Agilent Eclipse XDB-C18 4.6X50 mm, 1.8 μm, with electrospray in negative mode (ESI-). The resulting spectra were processed, and the analytes were identified and counted using multiple reaction monitoring (MRM) transition detection from the MS/MS fragmentation spectral library. The analytical parameters considered in the analysis of the substance of interest are described in Table 1.

To understand the possible biochemical alterations and health status of the organisms, the biomarkers evaluated in the gills and digestive glands (oyster) or gills and hepatopancreas (crabs) were as follows: ethoxyresorufin-O-deethylase (EROD) activity [36], dibenzylfluorescein dialquilase (DBF) [37] activity, glutathione S-transferase (GST) [38] activity, glutathione peroxidase (GPx) activity [39], reduced glutathione (GSH) levels [40], lipid peroxidation (LPO) levels [41], and DNA strand-break levels [42]. Acetylcholinesterase (AChE) activity in specimen muscles was also evaluated using the method by Ellman [43], with modifications by Herbert et al. [44] to use a microplate. Biomarker responses were standardized by total protein levels using the method proposed by Bradford [45] for different fractions of each tissue.

In order to evaluate lysosomal membrane stability, the hemolymph was withdrawn and hemocytes were assessed according Mártinez–Goméz et al. [46]. In the case of *U. cordatus*, the modifications proposed by Duarte et al. [32] were also applied.

The outliers present in the biomarker results were removed using the Grubbs test. After this step, the normality and homoscedasticity of the data were confirmed using the Shapiro–Wilk test and Bartlett’s test, respectively.

Next, the data were assessed using permutational multivariate analysis of variance (PERMANOVA) with paired analysis. Variance homogeneity was analyzed using PERMDISP within the PRIMER software, version 6.0.

To better understand the results, biomarker data for each species were integrated using the enriched integrated biomarker response (EIBR) system [47], with the data matrix values normalized for each biomarker and multiplied by the weight assigned to each biomarker according to its systemic importance (EROD, DBF, GST, GPx, and GSH = 1; LPO, DNA damage, and AChE = 2; NRRT = 3). Subsequently, all biomarker scores were summed for each treatment and for each tissue and divided by the sum of the weights to provide a final grade of effect for each treatment.

## 3. Results and Discussion

**Figure 1:**
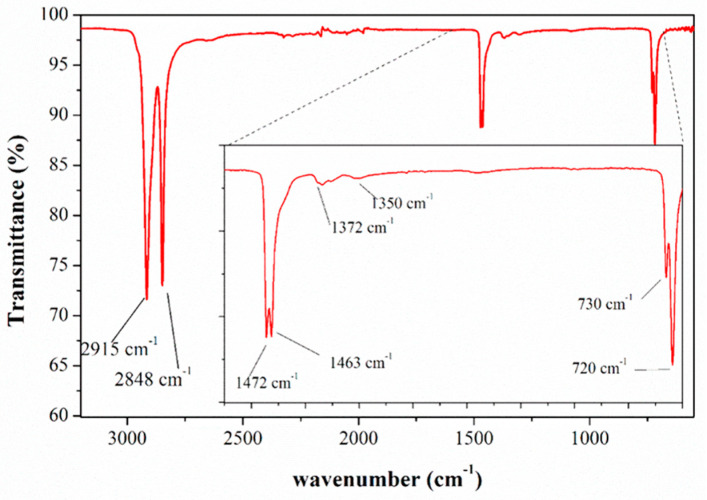
FT-IR (ATR) spectrum of LLDPE.

From the FT-IR (ATR) analysis, the polymer was confirmed as linear low-density polyethylene (LLDPE), as shown in Figure 1.

### 3.1. EE2 Quantification

Regarding microplastic contamination by EE2, a concentration of 35.40 µg·g^−1^ of EE2 was determined, with a recovery of 79.65% of the nominal concentration. Previous studies have found that ethinylestradiol tends to exhibit high adsorption by microplastics made from different polymers such as polyethylene and polyvinyl chloride [14,19,20], as well as those made of polyamide [48]. In the virgin microplastic (MP), EE2 was not detected (Table 2).

In this study, EE2 exhibited affinity for polyethylene-based microplastics, which exhibited high adsorption. This finding is due to the adsorptive behavior of EE2, which, unlike some substances, does not exhibit changes in its ability to associate with microplastics based on salinity [19,20]. Polymer-based features, such as structural characteristics, may also influence sorption behavior, since polyethylene presents a larger surface area compared to other polymers [49]. Particle size also has a direct influence on sorption behavior. In the study performed by Lu et al. [19], PVC particles that were 0.11 mm in size adsorbed more hormones than microplastics that were 4.7 mm in size. These results showed that the greater the particle’s coefficient of distribution, the greater its adsorption with the higher degree of irregularity.

The adsorption behavior of EE2 may be characterized by three phases. The first phase consists of rapid adsorption, followed by delayed adsorption, and finally, equilibrium. This process depends on the saturation of microplastic binding sites [20] and does not allow the microplastic to completely adsorb the substance.

In the MPE treatment group, organisms were able to accumulate EE2 (values between LOD and LOQ) even when levels were below the limit of detection in water (Table 3). This likely occurred because microparticles released this hormone into the experimental environment. EE2 concentrations in the microplastics at the end of the experiment were 0.66 µg·g^−1^, a value much lower than the amount measured at the beginning of the experiment (35.40 µg·g^−1^). The results of the *U. cordatus* assays differed slightly. Though EE2 had accumulated in the organisms (0.01401 µg·g^−1^) and decreased in the microplastics (1.284 µg·g^−1^) by the end of the experiment relative to the beginning, EE2 was detected in the water samples after the 7-day exposure period. EE2 concentration in the water was 1.93 µg·L^−1^. This result suggests that polyethylene microplastics serve as carriers and dispersers of pharmaceuticals and personal care products (PPCPs).

The ability of microplastics to interact with their surrounding environment and biota through sorption and desorption processes has been reported previously [17,50,51]. The details of this interaction vary depending on the substances involved. Lu et al. [21] found that EE2 tended to desorb approximately 49% of its PVC particle concentration; this ability was found to be directly related to its degree of hydrophobicity. However, the low concentrations and, in some cases, the absence of EE2 in the water samples after 7 days of exposure may be associated with EE2 degradation by light or biological processes that could reduce its concentration in the matrices.

EE2 detection in the tissues of the organisms tested is mainly due to the lipophilicity of the substance, as well as the transport potential of microplastics. A previous study evaluating the exposure of invertebrates to EE2 in water demonstrated that oysters of the species *C. virginica* exposed to 1 µg·L^−1^ of EE2 for 10 days exhibited high concentrations of the hormone after 1 day of exposure; however, after 5 days of exposure, EE2 was no longer detected in tissues [52]. Although the concentration of EE2 in MPs is higher than reported by this study, microplastics tend to desorb this substance gradually, thus providing continuous sources of contamination for the organisms that come into contact with them.

### 3.2. Biomarkers

The PERMANOVA results on *C. gasar* (Oyster) pointed out the time effect on all three treatments. The interaction between time and treatment revealed that after 3 days of exposure, all of the treatments differed from each other significantly. However, after 7 days of exposure, only the MPE group differed significantly from control MP groups (Table 4).

*U. cordatus* (crab) also demonstrated an influence of the time factor on all of the tested groups. After 3 days, the time vs. treatment interaction revealed significant differences only between the control group and the MPE group. After 7 days, the control group differed significantly from the MP group and the MPE group (Table 4).

**Figure 2:**
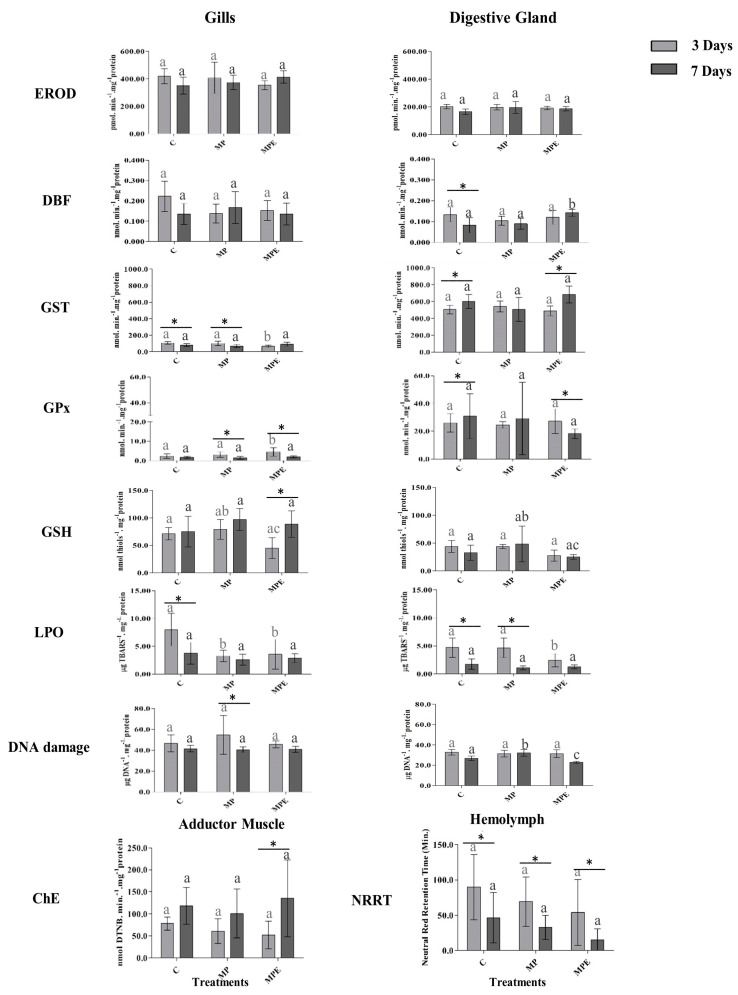
Mean and standard deviation of *Crassostrea gasar* biomarkers in different tissues and with different treatments after 3 days (light gray bars) and after 7 days (dark gray bars) of exposure. Light gray letters reflect differences between 3-day treatments, while dark gray letters reflect differences between 7-day treatments. Different letters represent significant differences between the treatments (*p* < 0.05). Asterisks (*) represent significant differences between the time periods in a given treatment (*p* < 0.05).

The biomarker results from *C. gasar* tissues (Figure 2) showed that after 3 days of exposure, GST activity in the gills was inhibited. GPx activity was induced in the MPE treatment group, though oxidative stress was not detected. In the digestive glands, an increase in DBF activity was observed in the MPE treatment group after 7 days of exposure. Additionally, the digestive glands revealed an increase in DNA strand breaks after 3 days of exposure to the MP treatment. Nevertheless, the neutral red retention time (NRRT) assay did not determine any significant differences between the treatments; a decrease in lysosomal membrane stability in the organisms exposed to the MPE treatment was observed over the course of the experiment.

According to PERMANOVA, the time factor was found to directly influence the biomarker responses evaluated. The oysters exhibited changes in phase 2 enzymes and the antioxidant system, oxidative stress in the gills, as well as worsened cell health in the hemolymph, particularly when exposed to the MPE treatment for 3 days; after 7 days of exposure, changes in the digestive gland (CYP3A-like activities), DNA strand breaks, and lysosomes in the hemolymph were also found in the hemolymph of organisms in the MPE group.

DBF activity in the digestive gland of *C. gasar* increased after exposure to the MPE treatment. These findings differ from those of Brandts et al. [53], who reported inhibited *cyp32* expression in the digestive gland of bivalve mollusks exposed to polystyrene nanoplastics combined with carbamazepine. The change observed in the current study may be linked to CYP3A-like metabolism of the hormone desorbed by the microplastic, since this enzyme is responsible for the biotransformation of this group of substances for subsequent excretion [37].

GST, an enzyme which corresponds to phase 2 of detoxification, was found to have inhibited activity in the MPE-treated oysters’ gills after 3 days of exposure. GST activity inhibition may be associated with an effect produced by the contaminated microplastics, or with increased activity of other enzymes performing detoxification [54].

In this study, the antioxidant system was represented by GPx and GSH. In the oysters’ gills, GPx activity was induced and GSH levels was increased after 3 days of exposure to the MPE treatment. Increased GPx activity is indicative of an increase in ROS production, since it is an important enzyme in the neutralization of basal levels of hydrogen peroxide [55]. GSH is known for being a key element in the transformation and excretion of contaminants, as well as in cellular antioxidant defense [56].

In the oysters, the assessment of oxidative stress through the measurement of thiobarbituric reactive substances revealed an absence of lipid peroxidation in the gills and digestive gland exposed to both the MP and MPE treatments after 3 or 7 days of exposure. These findings corroborate with previous studies in which mussels exposed to both spiked and virgin microplastics did not exhibit LPO [57,58]. It is believed that bivalves exposed to PPCPs tend to decrease their metabolism to increase their energy reserves, thus decreasing lipid peroxidation and minimizing oxidative damage [59].

In the analysis of DNA strand breaks, only digestive gland exposure to MPs after 7 days showed increased damages. Avio et al. [58] reported an increase in DNA strand breaks in the digestive tissue of bivalve mollusks caused by exposure to polyethylene particles. Meanwhile, the significant decrease in strand breaks that was observed after a longer exposure period (7 days) may be associated with the presence of EE2, since this hormone has been found to decrease DNA fragmentation in polychaetes exposed to different concentrations [7]; these prior results combined with the findings reported herein demonstrate that the presence of contaminants in microplastics may lead to effects that differ from those of microplastics in isolation.

No significant differences were found in the AChE activity and NRRT in the muscle and hemolymph of the oysters, respectively. However, it is possible to note a reduction trend after 3 days for AChE activity and after 3 or 7 days for the NRRT, demonstrating a disturbance in the organisms’ health throughout the assay.

**Figure 3:**
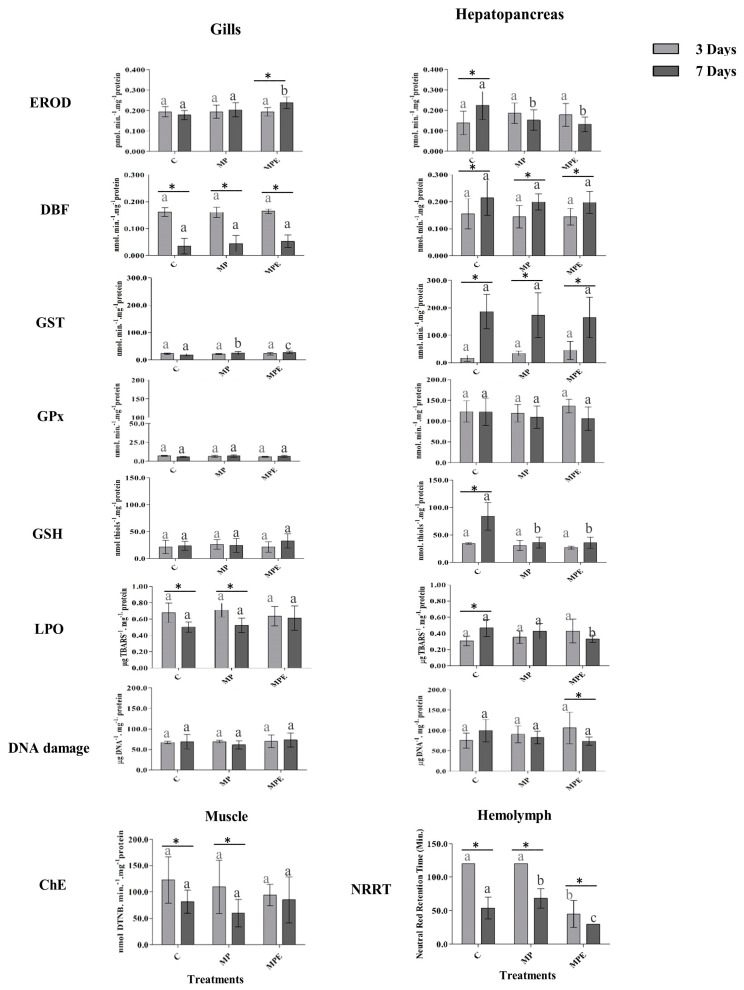
Mean and standard deviation of *Ucides cordatus* biomarkers in different tissues and with different treatments after 3 days (light gray bars) and after 7 days (dark gray bars) of exposure. Light gray letters reflect differences between 3-day treatments, while dark gray letters reflect differences between 7-day treatments. Different letters represent significant differences between the treatments (*p* < 0.05). Asterisks (*) represent significant differences between the time periods in a given treatment (*p* < 0.05).

The assessment of the *U. cordatus* biomarkers is detailed in Figure 3. After 7 days of exposure, the gills revealed an increase in EROD activity in the MP treatment group and increased GST activity in the MP and MPE treatment groups. In the hepatopancreas, EROD activities and GSH levels were inhibited in the MP and MPE treatment groups. The lysosomal membrane stability of the MPE treatment group differed significantly from the controls (*p* ≤ 0.05) after both the 3 and 7-day exposure periods. The organisms displayed distinct biochemical responses across the analyzed tissues (gills, hepatopancreas, and hemolymph), with effects observed in the MP and MPE treatment groups after 7 days of exposure.

EROD levels increased in the gills of the crabs exposed to the MPE treatment; this increase in the gills may be linked to the presence of EE2, as has been reported by Maranho et al. [9]. The authors found an increase in EROD in polychaetes exposed to different concentrations of EE2 and that this metabolic pathway is one of the main methods of EE2 degradation. However, in the present study, EROD levels decreased in the hepatopancreas in both the MPE and MP treatment groups. According to Anbumani and Kakkar [60], EROD activity can be inhibited in the hepatopancreas given the fact that microplastics are known to alter cytochromes P450 and may decrease their activity; they are therefore capable of producing more severe changes, such as cell death.

Although there are studies demonstrating an association between microplastic exposure and both increased and inhibited EROD activity in marine invertebrates [61,62], it is important to note that the exact mechanisms by which microplastics affect EROD activity in invertebrates are not fully understood, because these responses are variable and depend on a series of factors, including the type and size of microplastics, the exposure concentration, associated compounds, the duration of exposure, and the species of invertebrate involved.

GST activity was induced in the crabs exposed for 7 days to the MP and MPE treatments. This induction may be associated with an increase in phase 1 enzymes involved in the metabolism of organic compounds for excretion. This response has also been observed by Jeong et al. [63] in marine copepods exposed to polystyrene microplastics, who reported an increase in ROS, followed by an increase in GST activity.

In the crabs, only GSH levels were decreased after exposure to the MPE and MP treatments. Its inhibition may indicate suppression of the antioxidant defense system and, more specifically, the excretion of free radicals formed in the biotransformation process, since it is a major cofactor for GST and GPx [64].

Lipid peroxidation was not found in crabs exposed to the MP or MPE treatments. Similar results were reported in a study on amphipods exposed to different concentrations of EE2 [9]. DNA strand-break levels were also similar for all treatments and durations.

Crabs exhibited significant differences in lysosomal membrane destabilization when exposed to the MPE treatment. Previous studies have shown that the association of MPs with other contaminants tends to decrease the stability of the lysosomal membrane, and this relationship is time-dependent [19,65,66]. In addition, synthetic hormones such as EE2 cause destabilization of the lysosomal membrane that can lead to increased levels of Ca^2+^, which is a mediator of the effects of estradiol [67].

The changes observed in the gills of the two species evaluated are primarily correlated with the fact that they were exposed to these contaminants in water, since the gills serve as the animals’ primary defense barrier against xenobiotics [68]. Meanwhile, the changes observed in the digestive gland and hepatopancreas may be associated with both the initial effects on the gills or with the ingestion of these contaminants, since these organs are responsible for metabolizing exogenous substances.

They have numerous epithelial tissues with blind endings, such as basophilic, and digestive cells, such as the lysosome, which, in addition to the functions of intracellular digestion of nutrients and antioxidant defenses, are the main organelles for the sequestration and detoxification of organic pollutants [69].

**Figure 4:**
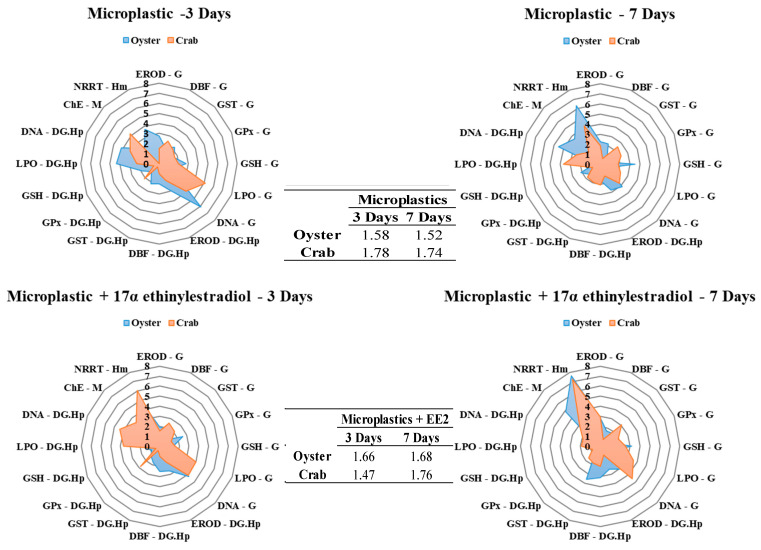
Biomarker response index in the gills (G), digestive gland (DG) or hepatopancreas (Hp), muscle (M), and hemolymph (Hm). The figure shows the behavior of the biomarkers in the control, microplastics, and microplastics + 17α ethinylestradiol treatments between species over time separately. The values presented between graphs refer to the physiological change score of each organism for the respective time and treatment.

Comparing the responses of the integrated biomarkers between the two species (Figure 3), it was possible to observe that crabs presented more significant biochemical disturbances than oysters, since the IBR indexes were always higher (excluding after 3-days, where crabs exposed to MPs showed alterations in DBF activity and LPO levels in the gills, and in AChE activity in the muscle, whereas oysters exhibited lower alterations in DNA damage in the gills and digestive gland, in LPO levels in the digestive gland, and in the NRRT). Regarding the 7-day period, crabs showed effects associated with DBF activity and LPO levels in the gills, LPO levels in the hepatopancreas, and the NRRT in the hemolymph, whereas in oysters, we observed DNA damage in the digestive gland and a lower NRRT. For the MPE experimental group, with the shorter exposure time (3 days), *C. gasar* demonstrated more biomarker responses, when compared to *U.cordatus,* associated with GPx and DNA damage in the gills, DBF activity and DNA damage in the digestive gland, and the NRRT in the hemolymph. After 7 days, there was an inversion between the species’ responses, since the crab showed the greatest changes associated with GST activity, LPO levels, and DNA damage in the gills, DBF, GST, and GSH activity in the hepatopancreas, and the NRRT in the hemolymph, as shown in Figure 4.

Taken together, our results reveal greater differences between the species exposed to MPEs. The biochemical and cellular changes may be directly linked to the species’ differing behaviors. *C. gasar* are filter feeders which are able to filter from 5 L to 25 L of water per hour [70], a process which allows for continuous exchange between the organisms and their environment, producing a high rate of depuration. Another relevant factor is that when under stress, bivalves tend to limit the amount of time that their valves are open, thus reducing their exposure to contaminants and forcing these animals to perform anerobic metabolism [52]. These behaviors may explain the reduced effects observed in oysters.

Because it is a semi-aquatic animal, *U. cordatus* comes into contact with both virgin and spiked microplastics in ways that differ from those of *C. gasar*. These oysters are exposed through their gills, while the crabs may ingest the particles or come into contact with them as they exchange water through their branchial chambers in order to maintain the necessary moisture levels to perform ion and gas exchange [31]. Although this study did not quantify microplastic ingestion rates, it was noted that shortly after inserting the crabs into the tanks containing microplastics, the individuals began to move their chelipeds, moving the water toward the oral region, opening and closing their oral apparatus (maxillipedes), and unwittingly ingesting microplastics in the surrounding water.

Overall, the metabolic responses of crabs and oysters exposed to microplastics, both virgin and adsorbed, are complex and may have significant implications for their health, fitness, and ecological roles. However, further research is needed to better understand why the metabolic consequences of microplastic exposure have a more pronounced impact on crabs. It is believed that the capacity for direct ingestion results in the accumulation of these particles in the digestive tracts and soft tissues of the animals, disrupting the digestion and metabolism of crabs and impairing their ability to absorb nutrients from food. Additionally, microplastics may have direct toxic effects on crabs, causing damage to the digestive tract and other organs, which can result in alterations in metabolism and feeding behavior in these animals [18,65,71,72,73].

Our results show that both species respond to the association of microplastics and EE2 by activating their detoxification systems. Subcellular effects were observed mainly through reduced lysosomal membrane stability, denoting impairments to global health status, which could lead to impacts at individual or population levels over time [18,74,75].

## 4. Conclusions

The microplastics demonstrated the capacity to adsorb 17α ethinylestradiol and serve as a carrier in aquatic environments, resulting in disruptions to species’ metabolism and sublethal effects on two mangrove species.

*C. gasar* showed effects within a short period of exposure (3 days) to the MPE treatment, with changes in the enzymes of phase 1 and 2, in addition to the antioxidant system.

*U. cordatus* showed effects after 7 days of exposure to the MPE treatment, with changes in the enzymes of phase 1 and phase 2, in the antioxidant system, in lipid peroxidation, and in the stability of the lysosomal membrane.

The invertebrates *C. gasar* and *U. cordatus* exhibited effects associated with both virgin and EE2-spiked microplastics, with more pronounced disturbances observed in crabs exposed to MPEs.

Our study presents initial evidence of the combined effects of polyethylene particles and 17α ethynylestradiol, underscoring the significance of assessing the impacts of microplastics as carriers of pharmaceuticals and personal care products in coastal areas.

## Figures and Tables

**Table 1:** Analytical parameters used in analyses of 17α ethinylestradiol (EE2) in microplastic, tissue, and water samples.

17α Ethinylestradiol (EE2): Analytical Parameters			
Level of Concentration of Analytical Curve (µg·L^−1^)	MRM Transition Counts	MLOD (µg·L^−1^)	MLOQ (µg·L^−1^)
4.687 to 300	279 > 133	0.12	0.40

MLOD: method limit of detection; MLOQ: method limit of quantification.

**Table 2:** Analytical recovery observed in substances in negative ion mode in spiked microplastics and 17α ethinylestradiol concentrations in uncontaminated microplastic (MP) samples and 17α ethinylestradiol-spiked microplastic (MPE) samples. The results are presented as µg·g^−1^.

	Analytical Recovery		
	Nominal Concentration (µg·g^−1^)	Measured Concentration (µg·g^−1^)	Recovery (%)
EE2	44.4	35.40	79.65
	**Microplastics before exposure**		
	EE2 (µg·g^−1^)		
MP	<MLOD		
MPE	35.40		

MLOD: method limit of detection.

**Table 3:** 17α ethinylestradiol (EE2) concentrations in the water, organism, and microplastic samples after exposure to a control treatment (C), a virgin microplastic treatment (MP), and an EE2-spiked microplastic treatment (MPE) in assays involving *C. gasar* and *U. cordatus*. The results are presented as µg·L^−1^ in the case of water and as µg·g^−1^ in the case of organisms and microplastics after exposure.

	Treatment	Water	Species	Microplastics after Exposure
		EE2 (µg·L^−1^)	EE2 (µg·g^−1^)	EE2 (µg·g^−1^)
*C. gasar*	C	<MLOD	<MLOD	-
	MP	<MLOD	<MLOD	<MLOD
	MPE	<MLOD	<MLOQ	0.66
*U. cordatus*	C	<MLOD	<MLOD	-
	MP	<MLOD	<MLOD	<MLOD
	MPE	1.93	0.01401	1.284

MLOD: method limit of detection; MLOQ: method limit of quantification; -: absent from sample.

**Table 4:** PERMANOVA calculated based on biomarker data from *Crassostrea gasar* and *Ucides cordatus*. Significant differences (*p* < 0.05) are in bold.

Main Test								
	***Crassostrea gasar***				***Ucides cordatus***			
	DF	MS	Pseudo-F	P (Perm)	DF	MS	Pseudo-F	P (Perm)
Time	1	101.82	8.8278	**0.001**	1	131.78	11.756	**0.001**
Treatment	2	32.486	2.8164	**0.001**	2	25.325	2.2592	**0.002**
Time vs. Treatment	2	36.979	3.206	**0.001**	2	35.009	3.1232	**0.001**
Pairwise—Time (T3 X T7)								
	***Crassostrea gasar***				***Ucides cordatus***			
	T	P (perm)	perms	P (MC)	T	P (perm)	perms	P (MC)
Control	2.4615	0.002	760	**0.002**	2.9181	0.002	742	**0.001**
MP	1.6692	0.006	755	**0.022**	2.243	0.001	753	**0.001**
MPE	2.8417	0.001	758	**0.001**	2.1195	0.001	758	**0.001**
Pairwise—Treatment								
	***Crassostrea gasar***				***Ucides cordatus***			
	T	P (perm)	perms	P (MC)	T	P (perm)	perms	P (MC)
3 Days of Exposure								
Control vs. MP	1.4699	0.018	752	0.059	0.86666	0.695	738	0.628
Control vs. MPE	2.1896	0.001	753	**0.001**	1.7069	0.009	776	**0.021**
MP vs. MPE	1.6532	0.017	749	**0.034**	1.3927	0.036	752	0.086
7 Days of Exposure								
Control vs. MP	1.2881	0.095	765	0.126	1.7032	0.009	771	**0.019**
Control vs. MPE	1.8678	0.001	755	**0.013**	2.2825	0.001	746	**0.001**
MP vs. MPE	1.9531	0.001	768	**0.009**	1.2866	0.103	749	0.154

## Data Availability

The dataset will be made available on request from the authors. The raw data supporting the conclusions of this article will be made available by the authors on request.

## References

[1] European Union (2013). Directive 2013/39/EU—European Parliament and of the Council, of 12 August 2013, Amending Directives 2000/60/EC and 2008/105/EC with Regard to Priority Substances in the Field of Water Policy. Off. J. Eur. Union.

[2] European Union Commission Implementing Decision (EU) 2018/840 of 5 June 2018 Establishing a Watch List of Substances to Be Monitored at Union Level in the Field of Water Policy, Pursuant to Directive 2008/105/EC of the European Parliament and of the Council, and Repealing Commission Implementing Decision (EU) 2015/495 [Notified under Number C (2018) 3362]. https://eur-lex.europa.eu/legal-content/PT/TXT/?uri=CELEX%3A32018D0840.

[3] Aris A.Z., Shamsuddin A.S., Praveena S.M. (2014). Occurrence of 17α-ethynylestradiol (EE2) in the environment and effect on exposed biota: A review. Environ. Int..

[4] Tang Z., Liu Z.H., Wang H., Dang Z., Liu Y. (2021). A review of 17α-ethynylestradiol (EE2) in surface water across 32 countries: Sources, concentrations, and potential estrogenic effects. J. Environ. Manag..

[5] Ying G.G., Kookana R.S. (2003). Degradation of five selected endocrine-disrupting chemicals in seawater and marine sediment. Env. Sci. Technol..

[6] Maranho L.A., Baena-Nogueras R.M., Lara-Martín P.A., DelValls T.A., Martín-Díaz M.L. (2014). Bioavailability, oxidative stress, neurotoxicity and genotoxicity of pharmaceuticals bound to marine sediments. The use of the polychaete Hediste diversicolor as bioindicator species. Environ. Res..

[7] Borysko L., Ross P.M. (2014). Adult exposure to the synthetic hormone 17α-ethynylestradiol affects offspring of the gastropods *Nassarius burchardi* and *Nassarius jonasii*. Ecotoxicol. Environ. Saf..

[8] Maranho L.A., Moreira L.B., Baena-Nogueras R.M., Lara-Martín P.A., DelValls T.A., Martín-Díaz M.L. (2015). A candidate short-term toxicity test using *Ampelisca brevicornis* to assess sublethal responses to pharmaceuticals bound to marine sediments. Arch. Environ. Contam. Toxicol..

[9] Silva A.Q., Abessa D.M.S. (2019). Toxicity of three emerging contaminants to non-target marine organisms. Environ. Sci. Poll. Res..

[10] Almeida Â., Silva M.G., Soares A.M., Freitas R. (2020). Concentrations levels and effects of 17alpha-Ethinylestradiol in freshwater and marine waters and bivalves: A review. Environ. Res..

[11] Eriksen M., Lebreton L.C., Carson H.S., Thiel M., Moore C.J., Borerro J.C., Galgani F., Ryan P.G., Reisser J. (2014). Plastic pollution in the world’s oceans: More than 5 trillion plastic pieces weighing over 250,000 tons afloat at sea. PLoS ONE.

[12] Kershaw P.J., Rochman C.M., GESAMP (2016). Sources, Fate and Effects of Microplastics in the Marine Environment: Part Two of a Global Assessment.

[13] Wu C., Zhang K., Huang X., Liu J. (2016). Sorption of pharmaceuticals and personal care products to polyethylene debris. Environ. Sci. Pollut. Res..

[14] Guo X., Wang J. (2019). Sorption of antibiotics onto aged microplastics in freshwater and seawater. Mar. Poll. Bull..

[15] Yu F., Yang C., Zhu Z., Bai X., Ma J. (2019). Adsorption behavior of organic pollutants and metals on micro/nanoplastics in the aquatic environment. Sci. Total Environ..

[16] Souza T.M., Choueri R.B., Nobre C.R., de Souza Abessa D.M., Moreno B.B., Carnaúba J.H., Mendes G.I., Albergaria-Barbosa AC R., Simões F.R., Gusso-Choueri P.K. (2023). Interactive effects of microplastics and benzo [a] pyrene on two species of marine invertebrates. Mar. Pollut. Bull..

[17] Zhu Z.L., Wang S.C., Zhao F.F., Wang S.G., Liu F.F., Liu G.Z. (2019). Joint toxicity of microplastics with triclosan to marine microalgae *Skeletonema costatum*. Environ. Poll..

[18] Nobre C.R., Moreno B.B., Alves A.V., de Lima Rosa J., da Rosa Franco H., Abessa D.M.S., Maranho L.A., Choueri R.B., Gusso-Choueri P.K., Pereira C.D.S. (2020). Effects of microplastics associated with triclosan on the oyster *Crassostrea gasar*: An integrated biomarker approach. Arch. Environ. Contam. Toxicol..

[19] Weis J.S., Alava J.J. (2023). (Micro)Plastics are Toxic Pollutants. Toxics.

[20] Porcino N., Bottari T., Mancuso M. (2023). Do really microplastics affects marine biota? A review. Animals.

[21] Lu J., Wu J., Wu J., Zhang C., Luo Y. (2021). Adsorption and desorption of steroid hormones by microplastics in seawater. Bull. Environ. Contam. Toxicol..

[22] Wu J., Lu J., Wu J. (2022). Effect of gastric fluid on adsorption and desorption of endocrine disrupting chemicals on microplastics. Front. Environ. Sci. Eng..

[23] Melo C.M., Silva F.C., Gomes C.H., Solé-Cava A.M., Lazoski C. (2010). Crassostrea gigas in natural oyster banks in southern Brazil. Biol. Invasions.

[24] Lüchmann K.H., Clark M.S., Bainy A.C., Gilbert J.A., Craft J.A., Chipman J.K., Thorne MA S., Mattos J.J., Siebert M.N., Schroeder D.C. (2015). Key metabolic pathways involved in xenobiotic biotransformation and stress responses revealed by transcriptomics of the mangrove oyster *Crassostrea brasiliana*. Aquat. Toxicol..

[25] dos Reis I.M., Mattos J.J., Garcez R.C., Zacchi F.L., Miguelão T., Flores-Nunes F., Toledo-Silva G., Sasaki S.T., Taniguchi S., Bicego M.C. (2015). Histological responses and localization of the cytochrome P450 (CYP2AU1) in *Crassostrea brasiliana* exposed to phenanthrene. Aquat. Toxicol..

[26] Catharino M.G.M., Vasconcellos M.B.A., Kirschbaum A.A., Gasparro M.R., de Sousa E.C.P.M., Minei C.C., Moreira E.G. (2015). Passive biomonitoring study and effect biomarker in oysters *Crassostrea brasiliana* (Lamark, 1819: Mollusca, Bivalvia) in Santos and Cananéia Estuaries in São Paulo State, Brazil. J. Radioanal. Nucl. Chem..

[27] Siebert M.N., Mattos J.J., Piazza C.E., de Lima D., Gomes C.H.A., de Melo C.M., Bainy A.C. (2017). Characterization of ethoxyresorufin O-deethylase activity (EROD) in oyster *Crassostrea brasiliana*. Comp. Biochem. Physiol. Part B Biochem. Mol. Biol..

[28] Melo G.A.S. (1996). Manual de Identificação dos Brachyura (Caranguejos e siris) do Litoral Brasileiro.

[29] Christofoletti R.A., Hattori G.Y., Pinheiro M.A.A. (2013). Food selection by a mangrove crab: Temporal changes in fasted animals. Hydrobiologia.

[30] Pinheiro M.A.A., Duarte L.F.A., Toledo T.R., Adams M.A., Torres R.A. (2013). Habitat monitoring and genotoxicity in *Ucides cordatus* (Crustace: Ucididae), as tools to manage a mangrove reserve in southeastern Brazil. Environ. Monit. Assess..

[31] Ortega P., Santos R.A., Lacouth P., Rozas E.E., Custódio M.R., Zanotto F.P. (2014). Cytochemical characterization of gill and hepatopancreatic cells of the crab *Ucides cordatus* (Crustacea, Brachyura) validated by cell metal transport. Iheringia. Série Zool..

[32] Duarte L.F.A., Souza C.A., Nobre C.R., Pereira C.D.S., Pinheiro M.A.A. (2016). Multi-level biological responses in *Ucides cordatus* (Linnaeus, 1763) (Brachyura, Ucididae) as indicators of conservation status in mangrove areas from the western atlantic. Ecotoxicol. Environ. Saf..

[33] Duarte L.F.A., Blasco J., Catharino M.G.M., Moreira E.G., Trombini C., Nobre C.R., Moreno B.B., Abessa D.M.S., Pereira C.D.S. (2020). Lead toxicity on a sentinel species subpopulation inhabiting mangroves with different status conservation. Chemosphere.

[34] Siegfried M., Koelmans A.A., Besseling E., Kroeze C. (2017). Export of microplastics from land to sea. A modelling approach. Water Res..

[35] U.S. Environmental Protection Agency (2007). Method. 1694: Pharmaceuticals and Personal. Care Products in Water, Soil, Sediment, and Biosolids by HPLC/MS/MSEPA821-R-08-002.

[36] Gagné F., Blaise C. (1993). Hepatic metallothionein level and mixed function oxidase activity in fingerling rainbow trout (*Oncorhynchus mykiss*) after acute exposure to pulp and paper mill effluents. Water Res..

[37] Gagné F., André C., Cejka P., Gagnon C., Blaise C. (2007). Toxicological effects of primary-treated urban wastewaters, before and after ozone treatment, on freshwater mussels (*Elliptio complanata*). Comp. Biochem. Physiol. Part C.

[38] McFarland V.A., Inouye L.S., Lutz C.H., Jarvis A.S., Clarke J.U., McCant D.D. (1999). Biomarkers of oxidative stress and genotoxicity in livers of field-collected brown bullhead, Ameiurus nebulosus. Arch. Environ. Contam. Toxicol..

[39] Sies H., Koch O.R., Martino E., Boveris A. (1979). Increased biliary glutathione disulfide release in chronically ethanol-treated rats. FEBS Lett..

[40] Sedlak J., Lindsay R.H. (1968). Estimation of total, protein-bound, and nonprotein sulfhydryl groups in tissue with Ellman’s reagent. Anal. Biochem..

[41] Wills E.D., Snell K., Mullock B. (1987). Evaluation of lipid peroxidation in lipids and biological membranes. Biochemical Toxicology: A Practical Approach.

[42] Olive P.L. (1998). DNA precipitation Assay: A rapid and simple method for detecting DNA damage in mammalian cells. Environ. Mol. Mutagen..

[43] Ellman G.L., Courtney K.D., Andres V., Featherstone R.M. (1961). A new and rapid colorimetric determination of acetylcholinesterase activities. Biochem. Pharm..

[44] Herbert A., Guilhermino L., Da Silva De Assis H.C., Hansen P.D. (1995). Acetylcholinesterase activity in aquatic organisms as pollution biomarker. Zeitschrift f. Angew. Zool..

[45] Bradford M.M. (1976). A rapid and sensitive method for the quantitationof microgram quantities of protein utilizing the principle of protein-dye binding. Anal. Biochem..

[46] Martínez-Gómez C., Bignell J., Lowe D. (2015). Lysosomal Membrane Stability in Mussels.

[47] Liu C., Chang V.W., Gin K.Y. (2013). Environmental toxicity of PFCs: An enhanced integrated biomarker assessment and structure–activity analysis. Environ. Toxicol. Chem..

[48] Lara L.Z., Bertoldi C., Alves N.M., Fernandes A.N. (2021). Sorption of endocrine disrupting compounds onto polyamide microplastics under different environmental conditions: Behaviour and mechanism. Sci. Total Environ..

[49] Teuten E.L., Rowland S.J., Galloway T.S., Thompson R.C. (2007). Potential for plastics to transport hydrophobic contaminants. Environ. Sci. Technol..

[50] Wang J., Tan Z., Peng J., Qiu Q., Li M. (2016). The behaviors of microplastics in the marine environment. Mar. Environ. Res..

[51] Vieira Y., Lima E.C., Foletto E.L., Dotto G.L. (2021). Microplastics physicochemical properties, specific adsorption modeling and their interaction with pharmaceuticals and other emerging contaminants. Sci. Total Environ..

[52] Brew D.W., Black M.C., Santos M., Rodgers J., Henderson W.M. (2020). Metabolomic investigations of the temporal effects of exposure to pharmaceuticals and personal care products and their mixture in the eastern oyster (*Crassostrea virginica*). Environ. Toxicol. Chem..

[53] Brandts I., Teles M., Gonçalves A.P., Barreto A., Franco-Martinez L., Tvarijonaviciute A., Martins M.A., Soares A.M.V.M., Delito L., Oliveira M. (2018). Effects of nanoplastics on Mytilus galloprovincialis after individual and combined exposure with carbamazepine. Sci. Total Environ..

[54] Subba M., Keough M.J., Kellar C., Long S., Miranda A., Pettigrove V.J. (2021). Potamopyrgus antipodarum has the potential to detect effects from various land use activities on a freshwater ecosystem. Environ. Poll..

[55] Trestrail C., Nugegoda D., Shimeta J. (2020). Invertebrate responses to microplastic ingestion: Reviewing the role of the antioxidant system. Sci. Total Environ..

[56] Huber P.C., Almeida W.P., Fátima Â.D. (2008). Glutathione and related enzymes: Biological roles and importance in pathological processes. Química Nova.

[57] Von Moos N., Burkhardt-Holm P., Kohler A. (2012). Uptake and effects of microplastics on cells and tissue of the blue mussel Mytilus edulis L. after an experimental exposure. Environ. Sci. Technol..

[58] Avio C.G., Gorbi S., Milan M., Benedetti M., Fattorini D., d’Errico G., Bargelloni L., Regoli F. (2015). Pollutants bioavailability and toxicological risk from microplastics to marine mussels. Environ. Poll..

[59] Freitas R., Coppola F., Costa S., Manzini C., Intorre L., Meucci V., Soares A.M.V.M., Pretti C., Solé M. (2019). Does salinity modulates the response of Mytilus galloprovincialis exposed to triclosan and diclofenac?. Environ. Poll..

[60] Anbumani S., Kakkar P. (2018). Ecotoxicological effects of microplastics on biota: A review. Environ. Sci. Poll. Res..

[61] Jong M.C., Li J., Noor H.M., He Y., Gin K.Y.H. (2022). Impacts of size-fractionation on toxicity of marine microplastics: Enhanced integrated biomarker assessment in the tropical mussels, *Perna viridis*. Sci. Total Environ..

[62] Wang T., Hu M., Xu G., Shi H., Leung J.Y., Wang Y. (2021). Microplastic accumulation via trophic transfer: Can a predatory crab counter the adverse effects of microplastics by body defence?. Sci. Total Environ..

[63] Jeong C.B., Kang H.M., Lee M.C., Kim D.H., Han J., Hwang D.S., Souissi S., Lee Sj Shin K.H., Park H.G., Lee J.S. (2017). Adverse effects of microplastics and oxidative stress-induced MAPK/Nrf2 pathway-mediated defense mechanisms in the marine copepod Paracyclopina nana. Sci. Rep..

[64] Nobre C.R., Moreno B.B., Alves A.V., Rosa J.L., Fontes M.K., Campos B.G., Silva L.F., Duarte L.F.A., Abessa D.M.S., Choueri R.B. (2022). Combined effects of polyethylene spiked with the antimicrobial triclosan on the swamp ghost crab (*Ucides cordatus*; Linnaeus, 1763). Chemosphere.

[65] Abouda S., Missawi O., Cappello T., Boughattas I., De Marco G., Maisano M., Banni M. (2022). Toxicological impact of environmental microplastics and benzo [a] pyrene in the seaworm *Hediste diversicolor* under environmentally relevant exposure conditions. Environ. Poll..

[66] Burlando B., Marchi B., Panfoli I., Viarengo A. (2002). Essential role of Ca^2+^-dependent phospholipase A2 in estradiol-induced lysosome activation. Am. J. Physiol. Cell Physiol..

[67] Arman S. (2021). Effects of acute triclosan exposure on gill and liver tissues of zebrafish (*Danio rerio*). Ann. Limnol. Int. J. Limnol..

[68] Faggio C., Tsarpali V., Dailianis S. (2018). Mussel digestive gland as a model tissue for assessing xenobiotics: An overview. Sci. Total Environ..

[69] Manzoni G. (2001). Textbook Ostras Aspectos Bioecológicos e Técnicas de Cultivo.

[70] da Silva L.F., Nobre C.R., Moreno B.B., Pereira C.D.S., de Souza Abessa D.M., Choueri R.B., Gusso-Choueri P.K., Cesar A. (2022). Non-destructive biomarkers can reveal effects of the association of microplastics and pharmaceuticals or personal care products. Mar. Pollut. Bull..

[71] Dos Santos C.C.M., Ferreira J.A., Dos Santos C.R.M., Amado L.L. (2021). Seasonal modulation of oxidative stress biomarkers in mangrove oyster (*Crassostrea gasar*) from an Amazon estuary. Comp. Biochem. Physiol. Part A Mol. Integr. Physiol..

[72] Dos Santos C.C.M., da Costa J.F.M., Dos Santos C.R.M., Amado L.L. (2019). Influence of seasonality on the natural modulation of oxidative stress biomarkers in mangrove crab *Ucides cordatus* (Brachyura, Ucididae). Comp. Biochem. Physiol. Part A Mol. Integr. Physiol..

[73] Moore M.N., Allen J.I., McVeigh A. (2006). Environmental prognostics: An integrated model supporting lysosomal stress responses as predictive biomarkers of animal health status. Mar. Environ. Res..

[74] Ringwood A.H., Hoguet J., Keppler C., Gielazyn M. (2004). Linkages between cellular biomarker responses and reproductive success in oysters–*Crassostrea virginica*. Mar. Environ. Res..

[75] Pereira C.D.S., Abessa D.M.S., Choueri R.B., Almagro-Pastor V., Cesar A., Maranho L.A., Martín-Díaz M.L., Torres R.J., Gusso-Choueri P.K., Almeida J.E. (2014). Ecological relevance of sentinels’ biomarker responses: A multi-level approach. Mar. Environ. Res..

